# Evaluation and acceptability of patient-reported outcome measures in women following pelvic organ prolapse procedures

**DOI:** 10.1186/s12913-023-09540-2

**Published:** 2023-06-13

**Authors:** Rasa Ruseckaite, Randi Jayasinghe, Claire Bavor, Joanne Dean, Oliver Daly, Susannah Ahern

**Affiliations:** 1 Department of Epidemiology and Preventive Medicine, Monash University, Melbourne, VIC 3004 Australia; 2 Department of Obstetrics and Gynecology, Western Health, Melbourne, VIC, 3000 Australia

**Keywords:** Pelvic floor disorders, Registry, Quality of life, Quality of care

## Abstract

**Background:**

The Australasian Pelvic Floor Procedure Registry (APFPR) captures clinical and surgical data in women undergoing pelvic floor procedures. The inclusion of patient reported outcome measures (PROMs) in the APFPR is a critical activity providing the additional patient perspective of their condition prior to surgery as well as monitoring beyond the usual post-surgical follow-up time. This study aimed to evaluate the acceptability of seven PROMs for women with pelvic organ prolapse (POP) and to determine the most suitable instrument for the APFPR.

**Methods:**

Semi-structured qualitative interviews were conducted with women with POP (n = 15) and their treating clinicians (n = 11) in Victoria, Australia. Interview topics covered appropriateness, content, and acceptability of seven POP-specific instruments identified through the literature to determine their suitability and acceptability for inclusion in the APFPR. We analysed the interview data using conventional content analysis.

**Results:**

All study participants agreed that PROMs were needed for the APFPR. Both women and clinicians suggested that some of the instruments were ambiguous, too long and confusing. The Australian Pelvic Floor Questionnaire was accepted widely amongst women and clinicians and recommended for inclusion in the APFPR. All participants agreed it would be appropriate to capture PROMs before surgery, and then followed up post-surgically. Email, phone call or postal mail-out were the preferred options for PROMs data collection.

**Conclusion:**

Most women and clinicians supported incorporating PROMs in the APFPR. Study participants believed that capturing PROMs would have potential use in individual care and improve outcomes of women with POP.

**Supplementary Information:**

The online version contains supplementary material available at 10.1186/s12913-023-09540-2.

## Background

Pelvic floor disorders, such as stress urinary incontinence (SUI) and pelvic organ prolapse (POP), are common, with prevalence increasing with parity and age. An increasing number of women have reported adverse events, such as erosion of mesh into the vagina or chronic pain, in response to procedures involving transvaginal mesh implants [1]. Many women have experienced significant suffering associated with complications and long-term health related quality of life (HRQoL) effects of pelvic floor mesh. Patient-reported outcome measures (PROMs) are important tools of assessing HRQoL, reported directly by the patient without interpretation by a clinician [2].

The Australasian Pelvic Floor Procedure Registry (APFPR) [3] captures the clinical and surgical data on women who had a pelvic floor procedure or mesh complications. The registry also has a consumer representative who takes part in the decision-making process on behalf of women participating in the registry. Capturing HRQoL data in the APFPR is very important for adding valuable information on safety and effectiveness of mesh-related procedures from a consumer perspective. However, a careful consideration is required for PROMs inclusion in the registry setting [4]. The length of the instruments and potential time burden should be considered [5].

A number of PROMs have been developed for women undergoing POP surgery [6, 7]. The aim of this study was to determine a suitable condition-specific PROM for the APFPR. This included assessing the relevance, clarity of wording, ease of use and clinical utility of selected instruments and the evaluation of preferred modes and methods for PROMs administration in the registry.

## Methods

### Study design

We employed a phenomenological approach and undertook semi-structured telephone interviews to understand viewpoints and opinions of the study participants. This approach, while focusing on aspects of a topic, does not confine participants to specific response categories defined in advance by researchers and is particularly appropriate when there is limited evidence on a phenomenon [8].

### Instruments

To select the available instruments that evaluated POP symptoms and their impact on HRQoL we reviewed the 6th International Consultation on Incontinence [9]. The Pelvic Floor Disability Index (PFDI-20), the Pelvic Floor Impact Questionnaire (PFIQ-7) [10], the Prolapse Quality of Life Questionnaire (P-QOL) [11], the Pelvic Organ Prolapse/Urinary Incontinence Sexual Questionnaire (PISQ-IR) [12] and the International Consultation on Incontinence Questionnaire Vaginal Symptoms Module (ICIQ-VS) [13] were included as they were ‘highly recommended’ by APFPR clinicians with published evidence of their validity, reliability and responsiveness to change. The Australian Pelvic Floor Questionnaire (APFQ) [14] and the Pelvic Floor Bother Questionnaire (PFBQ) [15] were included as they were also ‘recommended’ with published evidence on their validity and reliability [9].

### Recruitment and data collection

We used a convenience sampling for this project. We recruited women study participants recruited through social media and Pelvic Floor Support groups. Women participants were also recruited through private clinician referrals by directly approaching the key clinicians in the APFPR network who perform POP procedures in the state. An advertisement with a description of the study and contact details was developed.

To invite Australian urologists, gynecologists and urogynecologists to participate in this study, a short advertisement with description of the study was e-mailed to the APFPR Steering Committee members. The APFPR Steering Committee members were invited to forward the invitation to their colleagues. The invitation email was followed-up two weeks later.

Potential participants who expressed interest in the research were sent an explanatory statement and a copy of the PROMs. All participants were informed about the aims of the study and confidentiality procedures. Women were offered a $20 gift voucher as a token for participation in the study.

Semi-structured interview guides with open-ended questions were developed. The guide included questions on preferred mode and method of PROMs administration (i.e., self-administered or interviewer-administered, paper-and-pencil questionnaire or telephone- or computer-assisted technologies), frequency of data collection, and appropriateness of seven POP-specific instruments (see Additional file 1). Follow-up questions and prompts were used to obtain rich data. Women were asked to report whether the proposed instruments related to their health issues. Clinicians were asked to consider the potential use of PROMs data in clinical consults.

All interviews were conducted on the phone by three experienced researchers (RR, CB and RJ).

### Data analysis

All interviews were audio-recorded and then transcribed. To ensure quality of the data, the transcripts were checked against the voice files. All participants were offered the opportunity to review their transcripts. The interviews with women lasted 40 min (range 22–68) and with clinicians, 34 min (range 18–59).

Two researchers (RR and RJ) analyzed the data. The transcripts were reviewed, and identifying quotes and words were grouped according to themes and sub-themes as they emerged from the interviews. The themes were extracted inductively after the data were collected. Data saturation was determined when no new information was generated from successive interviews.

The data was analyzed using NVivo software (Version 12, QSR, Australia).

## Results

### Study design

Fifteen Caucasian women with surgical management of POP, mean age of 64.9 years (range 49–80) were interviewed (Table 1). Seven women reported having no complications with the mean duration since their last procedure being 6.5 years that ranged from 1 month to 15 years.

**Table 1:** Characteristics of women participants

Code	Age (years)	Highest Education	Time since the last procedure (years)	Complications
P001	52	Year 12	10	Yes
P002	60	Certificate IV	5–6	Yes
P003	78	Technical Education	0.08	No
P004	69	Tertiary	14	Yes
P005	55	Master’s degree	9	No
P006	54	Tertiary	9	Yes
P007	65	Tertiary	9	Yes
P008	49	Tertiary	3	Yes
P009	80	Tertiary	10	No
P010	74	Year 12	0.2	No
P011	70	Tertiary	6	No
P012	78	Intermediate certificate	4	No
P013	71	Intermediate certificate	0.4	Yes
P014	63	Diploma	2	No
P015	55	Year 10	15	Yes

Eleven Caucasian clinicians, consisting of ten specialists (general gynecologists or urogynecologists or obstetrician surgeons and gynecologists) and one nurse consultant participated in the study (Table 2).

**Table 2:** Clinician characteristics

Code	Years in practice	Number of POP procedures per year	Specialty
C001	19	19	Urology
C002	31	50	Urogynecology
C003	Not applicable	Not applicable	Research Nurse
C004	19	10	Urogynecology/Obstetrics
C005	17	50–60	Urogynecology
C006	Not provided	Not provided	Gynecology Registrar
C007	4	20–30	Urogynecology
C008	20	75	Gynecology
C009	27	50–100	Urogynecology
C010	26	50	Urology
C011	7	100	Urogynecology

### Evaluation of the instruments

#### The PFDI-20 instrument

In general, women with POP thought that the PFDI-20 was easy to complete, had a good choice of items with a satisfactory layout. However, some clinicians did not think that the PFDI-20 may be as relevant as it did not capture all patient experiences: “*I get a lot of people who’ve got urinary systems, overactive bladder, for example, which this doesn’t include and probably should. Because one of the treatable causes of overactive bladder is POP, so in that sense it’s a little bit deficient*” (C010).

In addition, some clinicians thought that some important topics were not covered: “*you need more on their pain, you need more on their sexual function*” (C004).

Women shared similar views, they also thought that this instrument would better suit if it was administered pre-surgery: “*Well, I think, it didn’t cover specifics. I would think a lot of it’s before you have surgery, a lot of this, this is all pre-surgical with regards to bulges, with regards to toileting, with regards to urinary leakage or incontinence. I think a lot of this is presurgical questionnaire*” (P014).

#### The PFIQ-7 instrument

Despite varying opinions most clinicians referred to the PFIQ-7 questions as straightforward and capturing patient experiences: “*I thought these questions were quite good”* (C003). Many women with POP expressed similar impressions: “it *seems to cover […] the ability to do physical activities, entertainment activities*” (P003). However, some other female participants believed that this instrument required improvement as it did not cover all the aspects of their health: “*I guess under the emotional health and the feeling frustrated. I think there, there could’ve been room to actually be able to voice why”* (P001). Women also felt that the PFIQ-7 did not cover all the aspects of work and social life: “*They don’t ask me how it impacted my work*” (P010).

A few clinicians said that a few questions lacked clarity or overlapped: “*I don’t think the questions are as clear as they should be*” (C006). Women agreed with this: “*I just thought the ‘entertainment’ one, like maybe the concert, and social activities outside the home, were sort of the same*” (P006).

#### The P-QOL instrument

Almost all study participants agreed that this instrument was easy and quick to complete as it covered all relevant aspects: “*It hits the bulk of the categories, in terms of they are good questions, they’re good bladder questions, good bowel questions*” (C002). Response options were acceptable for both groups of participants; however, the scoring system was not preferred by some clinicians: “*I think four is not a great scoring system. You’re better of having five. But it forces them not to tick the middle one*” (C005).

However, this instrument appeared too lengthy: “*When I printed out all the pages I thought, “Oh no, I’m not going to print all this stuff”* (P010). This reflection was supported by the clinicians: “*I think it’s very long. And if it was to be incorporated and used in other questionnaires, I think that would make the whole thing too long*” (C004).

#### The PISQ-IR instrument

Most clinicians believed that this instrument was relevant, however, it missed an important aspect of pain: “*What I find is that pain with intercourse is the thing that most people – if surgery affects them, it will be to cause pain with intercourse*” (C005). Clinicians also said that this instrument did not capture prolapse-specific symptoms: “*It doesn’t just ask that specific symptoms of the prolapse like urine and bowel symptoms that are given a standalone”* (C006).

Women also thought that this instrument was relevant, but noted that, it did not specify a timeframe nor reason for those not sexually active due to the procedure: “*Because I wasn’t sure […] ‘why are you not sexually active?’ ‘No interest’, ‘no partner’. It doesn’t say about a timeframe, but basically, the breakdown of my marriage was because my husband wanted a physical relationship, which I just couldn’t.”* (P006).

Overall, the study participants thought that this instrument would be hard to complete and some found it invasive.

#### The international consultation on incontinence questionnaire vaginal symptoms module (ICIQ-VS)

Both women and clinician participants felt that the ICIQ-VS captured most of patient experiences, however, it was not very comprehensive: “*It does cover the emotional side, the impact on sex and relationships and then everyday life, but it’s not as comprehensive*” (C006). Some clinicians thought that questions were ambiguous, especially those relating to sex life: “*Probably the harder ones for people to interpret are the sexual matters one. Not the, do you have a sex life, but do you worry about it and does it affect your relationship, or do you feel like your sex life’s been spoilt by vaginal symptoms? They’re the probably slightly more ambiguous ones*” (C001). Women also felt that some of the questions were hard to answer: *“[…] How much does it bother you,” those questions were a bit harder to answer”* (P004).

In addition, some clinicians were concerned with the scale: “*It’s about them, and it is useful in terms of before and after*” (C002).

Clinicians also thought that this instrument was too long and would make respondents frustrated: “*I feel this one would take me […] so it’s going to take a little bit longer. Whereas this one, you get asked the question, you have to answer it, and then you have to think some more about it. And then you go through – it’s like surveys like this – I virtually find frustratin*g” (C006).

#### The australian pelvic floor questionnaire (APFQ)

Most of the study participants found this instrument easy and simple: “*I mean the questions that are being asked here are really the questions that pretty much I ask in my language as well. I like that it’s Australian. And the questions are simple*” (C005). Women thought that it was relevant to their procedure and covered their experiences: “*Very relevant to the experience that I’ve been having because I’ve been having a lot of bladder problems*” (P004). They also felt that it was the most comprehensive compared to the other surveys: “*I thought it was a summary of quite a few of the other questionnaires. And I actually thought it was quite good, because it gave you a chance to write what you thought or the reason why you sort of answered like this*” (P006). This was seconded by the clinicians: “*It’s probably the best overall in terms of not missing things*” (C002).

When asked whether additional questions were required, a few participants thought that the instrument lacked details regarding urinary tract infections and bowel issues: “*The only thing it misses, and so do a lot of others, are urinary tract infections*” (C002); or “*The bowel function.”* (P009).

Despite that the APFQ appeared to be lengthy, the clinicians found it comprehensive, as it covered most of the aspects of health: “*I like they were short, sharp questions, it is a little bit longer but it’s quite comprehensive because it covered a lot of systems. And it’s clearly going through sexual function as well*” (C006). Women with POP did not mind the length:” *It wasn’t a problem. There were questions there that always have to be answered and so that you can see how things are all going together.*” (P012). However, one clinician thought that scoring was complex: “*In fact, this would have to be one of my favourite questionnaires, but the two weaknesses are that it is quite long − 43 questions - and the other thing is the scoring. This one is quite complex to score*” (C008).

#### The pelvic floor bother questionnaire (PFBQ)

Many participants believed that this instrument was useful. However, according to some clinicians the questions and response options were “*just not comprehensive enough*” (C006). They also believed that the instrument did not capture “*the emotional aspect*” or “*mental health and [women’s] daily lives*” (C007).

Women thought that this instrument was very general: “*it didn’t cover it to enough depth for me*” (P006). They suggested including additional questions regarding pain and relationship: “*Does pain or discomfort curtail your ability –’ and […] ‘Has this impacted your relationship? Because the amount of divorces within the surgically mesh injured is phenomenal*” (P007). Some women also found it hard to complete: “*There were a few questions I couldn’t answer a yes or a no to and there was only a yes or a no answer*” (P001).

In general, this instrument appeared to be the least preferred amongst the study participants.

#### Modes, methods, location and frequency for PROMs data collection

In general, most women preferred online administration of PROMs, however, they also suggested other options, such as postal mail-out or phone interviews: “*Well, I did print it out because I wear bifocals and doing a survey like this online without being able to sit down and consider the questions, I find it difficult”* (P011).

Clinicians thought that to ensure higher response rates PROMs should be collected at clinic: *“I’d probably do them in the clinic, as I said, you know, feasibility-wise, doing them at the time of consenting for the surgery is probably best. Perhaps doing it around the time of seeing the doctor because then they can ask any questions. Doing it at home, you know, I think there’s less likelihood that they’ll complete it. And so they don’t have the opportunity for that support”* (C011).

For the pre-operative questionnaire, some clinicians felt it would be best if patients filled them out at home: “*I feel it’s got to be filled in before they see the doctor and I think it would be best to send it out for them to fill out at home. […] I think that if you leave it to the clinic appointment, then some people might just be running late*” (C008). Women did not have preference but also thought that home location would be better: “*Either or, either or – probably I would be good doing it at home. I’d be good if they gave it at the clinic as well*” (P005).

Most women participants believed that collecting PROMs every three to six months would suit them best. In general, women agreed that frequent data collection was useful: “*I would go the six weeks, which is basically looking for any surgical related complications, and then three months – or maybe, you could flag the three months, and do six months – annual – depending on the responses*” (P006).

#### Potential barriers to completing PROMs

Some women participants were not certain how long it would take to complete the questionnaires: “*[…] I don’t know whether it would take anybody else long.”* (P003).

Some other women noted that some questionnaires were repetitive and that only one instrument should be used instead: “So *that, maybe, you could have the same questionnaire, but one – or had straight prolapse, you know.*” (P006).

Other barriers to completing PROMs included health literacy and physical issues: “*Health literacy. English not as their first language. Eyesight issues. You might have to have people like sometimes we’ve had to read the questions out to people and do it by telephone when we’ve had to complete data* […]” (P009).

## Discussion

We conducted qualitative interviews with women who had a surgical procedure for POP and clinicians experienced in treating pelvic floor disorders to determine the acceptability of including POP-specific PROMs in the APFPR. All participants believed that it would be beneficial to capture PROMs in the registry for future management of patient outcomes.

PROMs are valuable tools and have been used extensively in research and clinical practice in urogynecology [16]. Hundreds of PROMs are available for use in the evaluation and screening of pelvic floor disorders and to measure the results of treatment [6]. PROMs in registries are becoming more widely available and increasingly being used for benchmarking purposes [4, 17, 18]. A number of gynecological surgery registries worldwide capture HRQoL data [7, 19–23]. PROMs data in the APFPR will provide additional information to support the safety monitoring of mesh-related adverse events about a participant’s condition prior to surgery as well as monitor them beyond the usual post-surgical follow up time period [24].

Our study participants agreed that PROMs should be simple, short and intuitively understandable. They also agreed that PROMs should cover all aspects of life and daily experiences. In general, all proposed instruments were well-accepted, however, clinicians and women had varying views towards the individual instruments. Although these questionnaires were well-accepted, most of them did not cover all aspects of pelvic floor dysfunction: bladder, bowel, prolapse, and symptoms of sexual dysfunction.

The APFQ evaluates all pelvic floor symptoms, including bladder, bowel, sexual function, prolapse symptoms, symptom severity, impact on the quality of life and discomfort in women with pelvic floor disorders [14, 25, 26]. Therefore, most participants thought that the APFQ should be included as it covered all the aspects of women’s daily life and health meeting the criteria for inclusion in the APFPR. This instrument has been validated in community-dwelling women for application by interview [14] and for self-application [25] and has found to be “*reliable, valid and responsive*”. The APFQ was originally developed and validated in the English language, and has been widely used with versions translated and adapted for application in Chinese, German, French, Spanish, Turkish, Arabic and Serbian [27–31]. It will be available for culturally and linguistically diverse people in the registry. Understanding views, needs and sensitivities of this rapidly growing population in Australia is necessary in order to provide them an appropriate care and assistance [32]. The APFQ has been chosen for piloting in the APFPR in established registry sites using various modes and methods of administration.

Both women and clinicians in our study believed that PROMs should be collected before the procedure and then followed up post-surgically. No difference in the responses between urologists, urogynecologists and gynecologists was observed. The preferred options for PROMs data collection included email, phone call and postal mail-out. Women with POP thought that electronic PROMs data would be easy to integrate with the APFPR; however, according to the clinicians, collecting PROMs using paper-based methods in the clinic may improve data completeness. Nevertheless, electronic administration of PROMs costs less, results in similar or faster completion times and reduces administration times [33].

The strength in this study lies in the adoption of a qualitative descriptive study design that uncovers the perspectives of women without interpretation from outsiders such as clinicians [34]. Another strength of this study was involving a large sample of women with pelvic floor disorders providing a positive influence and further evidence on PROMs face validity for all of the instruments selected in our study [35].

This study therefore provides important insight into their preferences and views for incorporating PROMs in the registry, which supports further implementation and utilization of the data.

There are however several limitations that should be acknowledged. First, participants self-selected for this study based on an advertisement. This may have captured a non-representative sample of women who are involved in research, and clinicians who are more experienced and used PROMs previously.

Second, the instruments selected for this study measured slightly different aspects of HRQoL. For example, the APFQ provides a comprehensive assessment of pelvic floor symptoms; assessing urinary, bowel, vaginal and sexual symptoms. Others such as the ICIQ–VS questionnaire are shorter, relating just to vaginal symptoms. The PISQ–IR is used for assessment of female sexual function in women with female pelvic floor disorders. Therefore, careful consideration is required before choosing the appropriate PROMs. These instruments must be able to detect changes in patient condition over time, as well as differences between patients and patient populations. Furthermore, to achieve optimal outcomes, a consensus between researchers and patients is required to determine the best instruments for capturing patient-reported symptoms, such as fecal incontinence, urinary incontinence, constipation, lower urinary tract symptoms, and sexual dysfunction [36]. The third limitation was the role of the researchers in qualitative study design. This may introduce researchers’ bias into the results providing an inaccurate representation of participants’ perspectives [37].

## Conclusion

In conclusion, most women with POP and clinicians believed that PROMs should be incorporated in the APFPR. A pilot registry study to test the feasibility and practicality of collecting PROMs data in women with pelvic floor dysfunction using various modes and methods of administration is underway and will be conducted at selected registry sites to identify barriers and facilitators for PROMs completion, data entry and use.

## Electronic supplementary material

Below is the link to the electronic supplementary material.

Supplementary Material 1

## Data Availability

All data generated and/or analysed during this study are included in this published article.

## Abbreviations

APFPR: Australasian Pelvic Floor Procedure Registry
APFQ: Australian Pelvic Floor Questionnaire
HRQoL: Health Related Quality of Life
ICIQ-VS: International Consultation on Incontinence Questionnaire Vaginal Symptoms
P-QOL: Prolapse Quality of Life
PFDI: Pelvic Floor Disability Index
PFIQ: Pelvic Floor Impact Questionnaire
PFBQ: Pelvic Floor Bother Questionnaire
PISQ-IR: Pelvic Organ Prolapse/Urinary Incontinence Sexual Questionnaire
POP: Pelvic Organ Prolapse
PROMs: Patient Reported Outcome Measures
SUI: Stress Urinary Incontinence
